# Estimating variation in surface emissivities of intertidal macroalgae using an infrared thermometer and the effects on temperature measurements

**DOI:** 10.1007/s00227-014-2429-3

**Published:** 2014-04-03

**Authors:** Kathryn L. Van Alstyne, Theresa K. Olson

**Affiliations:** Shannon Point Marine Center, Western Washington University, 1900 Shannon Point Road, Anacortes, WA 98221 USA

## Abstract

Accurate measurements of surface temperatures with an infrared (IR) thermometer require input of the emissivities of the surfaces being measured; however, few determinations of the emissivities of intertidal organisms’ surfaces have been made. Emissivities of intertidal macroalgae were measured to determine whether algal species, measurement angle, hydration, and layering affected them. Emissivities were similar and averaged 0.94 among 11 of 13 species. The species with lower and more variable emissivities (*Chondracanthus exasperatus* and *Desmarestia viridis*) differed in morphology from the other species, which were relatively flat thin blades with little surface texture. Measurement angle caused emissivities to decrease significantly in *Mazzaella splendens* but not in three other species. Hydration and layering of *Ulva lactuca* also had no effect. At 22 °C, measured temperatures were within 1 °C of actual temperatures when thermometer emissivity settings ranged from 0.75 to 1.00. When emissivities were set lower than actual values, measured temperatures were lower than actual temperatures at 15 °C and higher than actual temperatures at 60 °C. When the IR thermometer was used to measure surface temperatures of nine species of intertidal algae immediately before they were inundated by the incoming tide, temperatures were higher in mid intertidal than low intertidal individuals and higher on a sunnier day than an overcast day. Temperatures of *U. lactuca* increased with increasing height on the shore, but temperatures of *Ulvaria obscura* did not. Temperatures were also higher in *Fucus distichus* blades than receptacles, and lower in *U. lactuca* and *M. splendens* occurring in the lower layers of stacks of algae.

## Introduction

Climate change is projected to result in a net increase in temperature worldwide; however, its effects on local intertidal environments and organisms are complex because temperatures during low tide are also affected by interactions between topography, air and water circulation, and the timing of the tides (Bell 1995; Helmuth and Hofmann 2001; Helmuth et al. 2002, 2010, 2011; Denny et al. 2011). Because of these multiple interacting factors, it is difficult to theoretically predict the temperatures that intertidal organisms will experience at any given location. Organisms inhabiting the intertidal zone can experience rapid changes in temperatures as a result of the convection of heat to and from the air as they become emersed during a low tide or to and from seawater when they become inundated during an incoming tide, especially if there are large differences in air and seawater temperatures (Helmuth et al. 2010). Temperatures at low tide are also affected by the organism’s morphology, evaporative heat losses, the absorption, or loss of heat via conduction from the surface they are on or via thermal radiation from the sun (Bell 1995; Broitman et al. 2009). As a result, the surface temperatures of emersed organisms can be substantially different from the temperatures of the surrounding air or water and these large fluctuations in temperatures can have numerous physiological and ecological consequences (e.g., Smith and Berry 1986; Bell 1993; Davison and Pearson 1996; Tomanek and Helmuth 2002; Dethier et al. 2005; Morelissen and Harley 2007; Williams et al. 2013).

In order to understand the effects of temperature on the physiology and ecology of marine organisms, empirical measurements of surface temperatures are needed. Researchers interested in questions about the responses of intertidal organisms to temperature have employed a variety of methods for quantifying it. Each of these methods has advantages and disadvantages, which are in part dependent on the specific question that is being addressed. Generally, measurements of temperatures experienced by the organisms are more useful than temperatures of the nearby environment (Gilman et al. 2006; Helmuth et al. 2010) because the temperatures that organisms experience can be a function of very small-scale microtopographic features of the environment coupled with morphological, physiological, and behavior mechanisms used by organisms to alter their internal environments (Harley and Helmuth 2003; Helmuth et al. 2010). For example, biomimetic devices such as “robomussels” and “robolimpets” have been successfully employed by invertebrate ecologists to quantify the thermal environment experienced by sessile intertidal invertebrates (Fitzhenry et al. 2004; Lima and Wethey 2009). They are useful because they can measure temperatures in locations and at scales that are relevant to the organisms over long periods of time and because they mimic the morphologies of the organisms. However, they are less useful for understanding the thermal milieu experienced by seaweeds. Seaweeds are typically, although not always, composed of relatively thin tissues that rapidly conduct heat across the whole organism; they also differ in tissue composition from shelled invertebrates enough that these devices may not provide accurate estimates of their temperatures.

The use of infrared (IR) surface temperature measurements provides an alternative method for measuring temperatures at very localized scales that can be appropriate for investigating the thermal environment experienced by intertidal macroalgae (Cox and Smith 2011). Infrared surface temperatures can be measured using both IR thermographic cameras and handheld thermometers (pyrometers), although pyrometers are more widespread because of their lower cost. Both thermographic cameras and pyrometers measure the thermal radiation emitted by an object ($j^{*}$). The reading is then corrected for the object’s emissivity (*ε*) to obtain an estimate of its surface temperature (*T*) using the equation:

$$j^{*} \, = \,\varepsilon \sigma T^{ 4}$$

where *σ* is the Stefen–Boltzmann constant (5.67 × 10^−8^ W m^−2^ K^−4^). A substance’s emissivity is the ratio of the energy radiated by it relative to the amount of energy that would be emitted by a black body of the same substance at the same temperature in equilibrium (Rogalski and Chrzanowski 2002). The emissivity is dependent on the object’s chemical composition as well as its physical structure. In order for temperature measurements taken with a handheld IR thermometer to be correct, the correct emissivity for the surface being measured has to be entered into the device, although many less expensive IR thermometers lack the ability to do this.

As the use of IR temperature sensors continues to rise, it will become increasingly important to have accurate emissivity measurements for a range of organisms. To our knowledge, emissivities of common temperate intertidal algae have yet to be quantified. Because there can be differences in physical structure or chemical composition among macroalgal species, their surfaces could radiate different amounts of energy, leading to errors in temperature measurements if differences in emissivities are not corrected for when using IR thermometers. To better understand how emissivities differ among temperate intertidal algae and evaluate the potential utility of IR thermometers for conducting rapid in situ surface temperature measurements for these organisms, we examined intraspecific and interspecific variation in the emissivities of macroalgal surfaces. We also compared emissivities of dry and damp algae, and of single pieces versus algae that are layered. We then used the IR thermometer to obtain in situ surface temperature measurements of intertidal macroalgae at multiple tidal levels immediately before they become submerged at two sites in western Washington State.

## Methods

### Algal collections

The algae that were used in this study were species that are abundant on rocky beaches and mud flats in the region. They included representatives of the three major macroalgal phyla found in intertidal habitats—red (phylum Rhodophyta), green (phylum Chlorophyta), and brown (phylum Heterokontophyta; Class Phaeophyceae) algae and had a diversity of thallus morphologies. They were collected from the beach in front of the Shannon Point Marine Center (48°31′N, 122°41′W) and from Ship Harbor (48°30′N, 122°41′W), about 1 km from the Shannon Point Beach. The Shannon Point Beach is a cobble beach that hosts a diversity of macroalgal species whereas Ship Harbor is a relatively homogeneous mud flat that is dominated by eelgrasses (*Zostera marina* and *Zostera japonica*) and several ulvoid algal species (Class Ulvales: *Ulva intestinalis*, *Ulva lactuca*, *Ulva linza*, and *Ulvaria obscura* var. *blytii*). Most algae were used immediately after collection, although some were retained in flow-through seawater tables for up to 2 days prior to being used to obtain emissivity values.

### Laboratory emissivity measurements

The emissivities of the surfaces of 13 species of algae (*N* = 5 individuals per species) were measured in a laboratory drying oven at 50 *°*C with a Fluke 572CF IR thermometer (Fluke Corporation, Everett WA) that allowed the user to set the emissivity manually. The 50 °C temperature was chosen based on Thermal Vision’s recommendation (http://www.thermalvision.ie/2012/01/measuring-emissivity/) that the temperature used to measure emissivity be at least 20 °C higher than the ambient temperature. The Fluke 572CF IR thermometer has a reported accuracy of the greater of 1 % of the reading or 1.1 °C at temperatures above 0 °C and a reported repeatability of the greater of ±0.5 % of the reading or ± 0.5 °C (Fluke 2013). Its operating temperature range is −30 to 900 °C. The thermometer generates laser beams that produce three aiming dots, which indicate the center and diameter of the circular field of view from which the measurements are obtained. The pyrometer’s field of view changes with the distance from the object. All of our measurements were taken so that the field of view was as small as possible, which put the end of the thermometer about 0.3 m from the object being measured and produced a field of view of about 1.2 cm^2^.

To determine emissivities, pieces of algae were attached with 2 cm × 12 cm pieces of Scotch 33 black electrical tape to a 15 cm × 15 cm × 2.5 cm aluminum block that was placed in a 50 °C drying oven that had been turned on its side and had its door open. The algal pieces were approximately 3 × 13 cm in size, although this varied somewhat by species. The aluminum block was used to maintain a constant temperature across the surfaces of the tape and the algae. To determine the emissivity of the algae, we first measured the surface temperature of the black electrical tape (which has a known emissivity of 0.95) using the IR thermometer with the emissivity set to 0.95. Then, we measured the algal surface temperature and adjusted the emissivity setting on the thermometer until the temperature reading of the algal surface matched the temperature reading of the tape. All measurements were done inside the drying oven. For the initial measurements, care was taken to make sure that the thermometer was aimed straight down on both the algae and the tape. Ten replicate measurements were made on each of five replicate pieces of algae per species. All of the initial measurements were made on fully hydrated pieces of algae that were one layer thick. An average was taken of the replicate measurements for each algal piece and the emissivities among species were compared with a Kruskal–Wallis test because the data could not be transformed in a way that would allow them to be normally distributed.

To examine the effects of the angle at which the thermometer was pointed at the surface, emissivity determinations were made as described above on *Mazzaella splendens*, *Nereocystis luetkeana*, *Saccharina latissima*, and *U. lactuca* with the thermometer at a 90° (*N* = 5–10 individuals) angle to the algal surface and at a 45° angle (*N* = 11–22 individuals). The effects of angle were compared for each individual species with *t* tests. The data for *S. latissima* were angularly transformed to meet the assumption of normality.

To determine the effects of algal tissue hydration, emissivity measurements were made as described above on *M. splendens*, *N. luetkeana*, and *U. lactuca*. The algae were either allowed to dry in the oven or kept hydrated by applying water to their surfaces (*N* = 5 individuals per treatment per species). After the data were angularly transformed to meet the assumption of normality, they were analyzed with a two-way ANOVA with algal species and condition (wet versus dry) as factors. A series of emissivity determinations were also made on *U. lactuca* as described above when the algal thalli were attached to the aluminum block singly, layered two blades thick, layered four blades thick, and layered eight blades thick (*N* = 5 per treatment). The effect of layering was compared with a one-way ANOVA.

To examine the effects of varying the thermometer’s emissivity setting on the temperature reading from an algal surface, we measured the surface temperatures of *U. lactuca* (*N* = 5) at emissivity settings ranging from 0.7 to 1.0 at increments of 0.02. These measurements were conducted in a 60 °C drying oven, at room temperature (22 °C), and outdoors (15 °C).

### Field measurements

To evaluate the utility of the handheld IR thermometer in the field and determine the temperature extremes that intertidal algae at these sites experience, measurements were made of algal surface temperatures at low tide in the intertidal zones of the Shannon Point Beach and Ship Harbor. In this region, the lowest summer low tides during spring tide series are typically in mid to late morning. Therefore, all surface temperature measurements were made immediately before the algae were inundated by the incoming tide so that the algae were exposed to sunlight for as long as possible before immersion.

At the Shannon Point Beach, measurements were made of surface temperatures of four algal species (*Fucus distichus* ssp. *edentatus*, the crustose “*Petrocelis*” stage of *Mastocarpus papillatus*, *Porphyra* sp., and *U. lactuca; N* = 5–10 individuals per species per day) in the mid intertidal zone at approximately 0.2 m above mean low water (MLW) and of five algal species (*Alaria marginata*, *M. splendens*, *N. luetkeana*, *S. latissima*, and *U. lactuca; N* = 5–10 individuals per species per day) in the low intertidal zone at approximately 0.6 m below MLW. All algae used for these measurements were either growing on the upper surfaces of small cobbles or lying across the cobbles. The measurements were made on July 22, 2013, when the skies were overcast, the air temperature was 13 °C, and the irradiance was 242 W m^−2^; they were made again on July 24, 2013, when the skies were clear, the weather was sunny, the air temperature was 16 °C, and the irradiance was 786 W m^−2^. Air temperature and irradiance data are from a Davis Vantage Pro™ weather station located on top of the roof of the Shannon Point Marine Center, about 75 m from the beach where the measurements were made. Separate two-way ANOVAs were conducted for the mid and low intertidal measurements with species and the date the measurements were taken as factors. The data were transformed with an angular transformation prior to the analyses.

At Ship Harbor, we tested the hypothesis that the surface temperatures of ulvoid macroalgae that are measured immediately before inundation by the incoming tide increase with the height on the shore at which the algae are found. To do this, we measured surface temperatures of individual pieces of *U. lactuca* and *U. obscura* as the tide was coming in on July 23, 2013, when the air temperature was 20 °C. Because these two species cannot be reliably distinguished in the field, we took measurements of surface temperatures of 85 individuals at tidal heights ranging from 0.65 m below MLW to 0.28 m above MLW, noted the time at which the measurements were made, and then collected a piece from each alga for later identification in the laboratory. The algal samples were transported to the Shannon Point Marine Center where they were identified by microscopic examinations of algal sections. The height on the shore of each algal piece was estimated by determining the height of the water at the time the alga was collected using Nobeltec Tides and Currents™ software. Separate Spearman’s rank correlation analyses were used to determine whether surface temperatures increased with increasing height on the shore for each species.

We also tested several hypotheses regarding the distributions and morphologies of intertidal algae using the IR thermometer at the Shannon Point Beach on July 22 and July 24, 2013. To determine whether being covered by a layer or layers of algae caused surface temperatures of algae to be cooler, we measured temperatures of *M. splendens* blades, which are typically about 0.5 mm thick, and *U. lactuca* blades, which are typically 50–100 μM thick, that were found layered in stacks on the beach. Starting from the top layer, we measured the temperature of each individual and then immediately pulled back that layer and measured the temperature of the next individual in the stack. The number of layers in these stacks of algae ranged from three to eight. We then subtracted the temperature of each alga in the lower layers from the temperature of the individual at the top of the stack and used the average values to test for a correlation between the temperature difference and the location of the individual in the stack.

Comparisons were also made between the blades and receptacles of *F. distichus* (*N* = 5 per tissue type per day) to test the hypothesis that the surfaces of the reproductive structures are cooler than surfaces of the non-reproductive blades of the plants. These measurements were made on both July 22 when it was overcast and on July 24 when it was sunny. A two-way ANOVA was used to analyze these data with tissue type and date as factors. To determine whether the upright gametophyte stage and the crustose “*Petrocelis*” sporophyte stages of *M. papillatus* had different surface temperatures, we measured the surface temperatures of both life history stages at a tidal height of 0.2 m above MLW (*N* = 5 individuals per stage). These measurements were compared with a two sample *t* test.

## Results

### Emissivity measurements

**Fig. 1:**
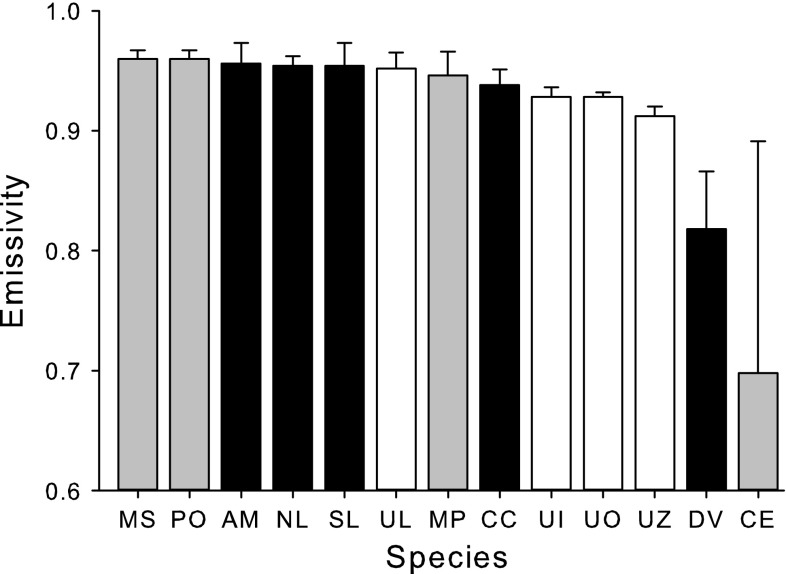
Emissivities of intertidal algal surfaces at 50° C (means ± 1 SD; *N* = 5–6). White bars are green algae (Chlorophyta), *gray bars* are red algae (Rhodophyta), and *black bars* are brown algae (Heterokontophyta, Phaeophyceae). MS: *Mazzaella splendens*, PO: *Porphyra* sp., AM: *A. marginata*, NL: *N. luetkeana*, SL: *S. latissima*, UL: *U. lactuca*, MP: *M. papillatus*, CC: *Costaria costatum*, UI: *U. intestinalis*, UO: *Ulvaria obscura*, UZ: *Ulva linza*, DV: *Desmarestia viridis*, CE: *Chondracanthus exasperatus*

Emissivities of algal surfaces differed significantly among species (Fig. 1; Kruskal–Wallis: *df* = 12, *H* = 45.11, *P* < 0.001). Two species, *Chondracanthus exasperatus* and *Desmarestia viridis*, had emissivities that were lower than the other algae examined. The rest clustered into another group and had emissivities that ranged from 0.91 to 0.96 and averaged 0.94.

**Fig. 2:**
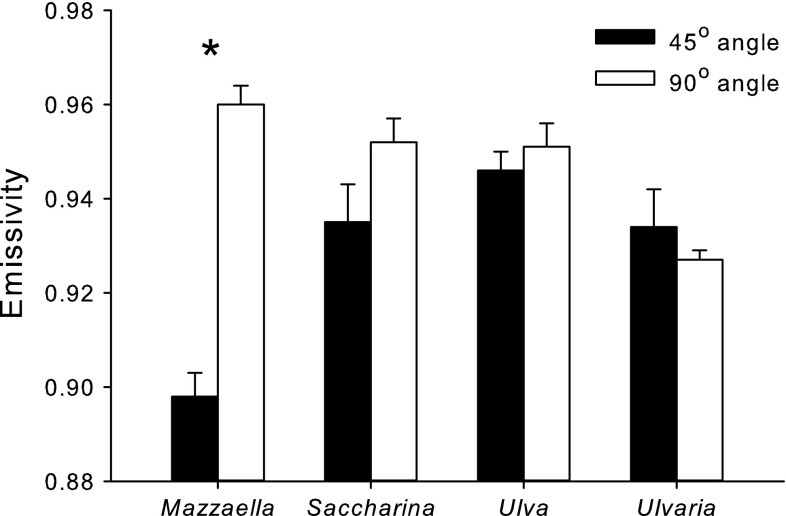
Emissivities of *U. lactuca, U. obscura, S. latissima, and M. splendens* surfaces at 50 °C (means ± 1 SE; *N* = 5–22) when the measurements were taken at a 45° (*black bar*) or 90° (*white bar*) angle. *Asterisks* indicate temperatures that are significantly different (*t* test, *P* < 0.05)

The effects of the angle at which the IR thermometer has held with respect to the algal surface significantly decreased (Fig. 2) the average emissivity of the surfaces of *M. splendens* (*t* test: *df* = 18, *T* = −9.27, *P* < 0.001); however, the measurement angle had no significant effect on emissivities of *S. latissima* (*t* test: *df* = 13, *T* = −1.75, *P* = 0.104), *U. lactuca* (*t* test: *df* = 9, *T* = −0.74, *P* = 0.477), or *U. obscura* (*t* test: *df* = 15, *T* = 0.82, *P* = 0.423).

When emissivities were compared among wet and dry *U. lactuca*, *M. splendens*, and *N. luetkeana*, there were no statistically significant differences among the three species (two-way ANOVA, species effect: *df* = 2, *F* = 0.69, *P* = 0.511) nor among wet versus dry algae (two-way ANOVA, condition effect: *df* = 1, *F* = 1.54, *P* = 0.226). Likewise, there was not a significant species × condition interaction (two-way ANOVA, species × condition effect: *df* = 1, *F* = 0.71, *P* = 0.500). The average emissivities (mean ± 1 SD) were 0.95 ± 0.011 (*N* = 10) for *U. lactuca*, 0.96 ± 0.014 (*N* = 10) for *M. splendens*, and 0.95 ± 0.017 (*N* = 10) for *N. luetkeana*. When comparing emissivities of layered *U. lactuca* with different numbers of layers, the number of layers had no effect on emissivity (one-way ANOVA: *df* = 3, *F* = 0.78, *P* = 0.524) and the average emissivity was 0.96 ± 0.008 (mean ± 1SE, *N* = 20).

**Fig. 3:**
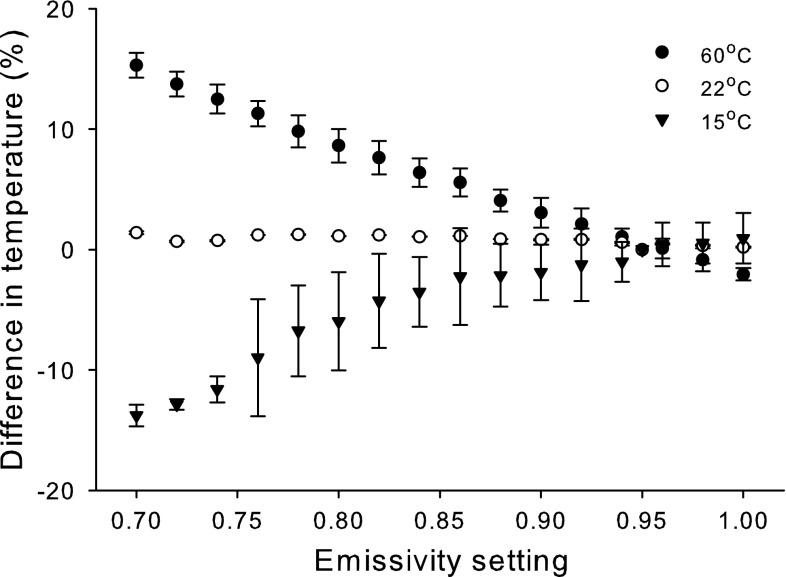
Differences between actual and measured temperatures (means ± 1 SE) of the surfaces of *U. lactuca* at 60 °C (*closed circles*), 22 °C (*open circles*), and 15 °C (*triangles*) obtained when the emissivity was adjusted by the user from 0.7 to 1.0

When the IR thermometer was set at emissivities ranging from 0.7 to 1.0, the differences between the actual and measured (indicated) temperatures differed depending on both the actual temperature and the difference between the emissivity entered into the thermometer (the set emissivity) and the actual emissivity of the algal surface (Fig. 3). At 22 °C, indicated temperatures were within approximately 1 °C of the actual temperature, even when the set emissivity was 0.25 lower than the actual emissivity. At 15 and 60 °C, the differences between the indicated and actual temperatures increased with increasingly incorrect emissivity settings. When the set emissivity was too low, the indicated temperatures were consistently lower than actual temperatures at 15 °C and higher than the actual temperatures at 60 °C. To get indicated temperatures that were within an average of 1 °C of the actual temperature, the set emissivity had to be within −0.01 and +0.05 of the actual emissivity at 15 °C; at 22 °C, the set emissivity had to be within −0.07 and +0.05 of the actual emissivity to get indicated temperatures that were within 1 °C of the actual temperature; at 60 °C, the set emissivity had to be within −0.01 and +0.03 of the actual emissivity to get indicated temperatures that were within 1 °C of the actual temperature.

### Measurements of surface temperatures in the field

**Fig. 4:**
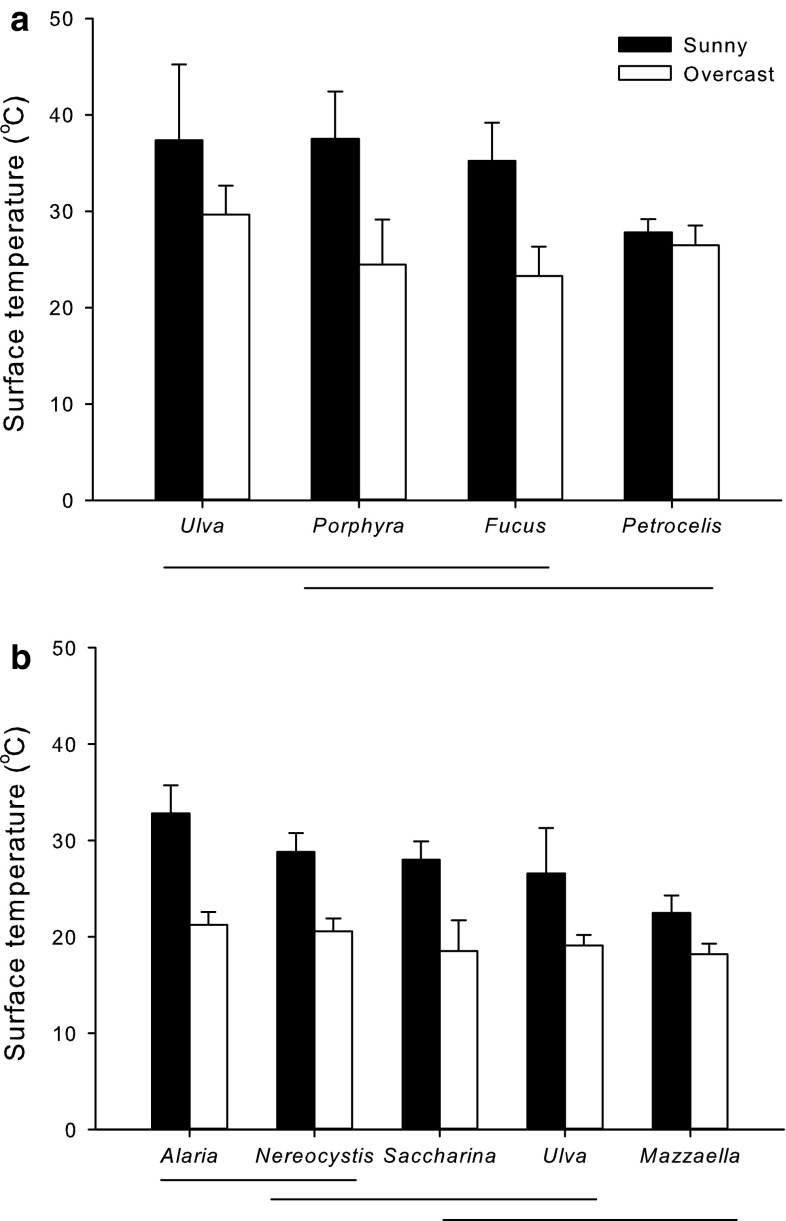
Mean surface temperatures (±1 SD, *N* = 10–15) of **a** the mid intertidal algae *U. lactuca*, *Porphyra* sp., *F. distichus*, and the “Petrocelis” stage of *M. papillatus*, and **b** the low intertidal algae *A. marginata*, *N. luetkeana*, *S. latissima*, *U. lactuca* and *M. splendens* on the Shannon Point Beach immediately before they were immersed by an incoming tide on a sunny (*black bars*) and an overcast day (*white bars*). Lines beneath the graphs indicate species for which means were not significantly different (Tukey’s test: *P* > 0.05)

Surface temperatures of macroalgae immediately before inundation by the incoming tide at the Shannon Point Beach differed among species and among days in both the mid and low intertidal zones (Fig. 4; Table 1). Temperatures were generally lower in species located in the low intertidal zone where the average algal surface temperatures on the sunny day ranged from 22.5 to 32.8 °C; in the mid intertidal zone, they ranged from 27.8 to 37.3 °C. On average, surface temperatures were 8.2 °C higher on the sunny day for the low intertidal species and 8.6 °C higher for the mid intertidal species.

**Table 1:** Results of two-way analyses of variance conducted on surface temperature measurements of macroalgae in the (a) mid and (b) low intertidal zones of the Shannon Point Beach

Factor	*df*	SS	MS	*F*	*P*
(a) Mid intertidal zone					
Species	3	0.049	0.016	3.74	0.018
Date	1	0.172	0.172	38.98	<0.001
Species × date	3	0.048	0.016	3.67	0.019
Error	43	0.189	0.004		
Total	50	0.458			
(b) Low intertidal zone					
Species	4	0.075	0.019	7.57	<0.001
Date	1	0.310	0.310	124.98	<0.001
Species × date	4	0.017	0.004	1.67	0.172
Error	49	0.121	0.002		
Total	58	0.523			

**Fig. 5:**
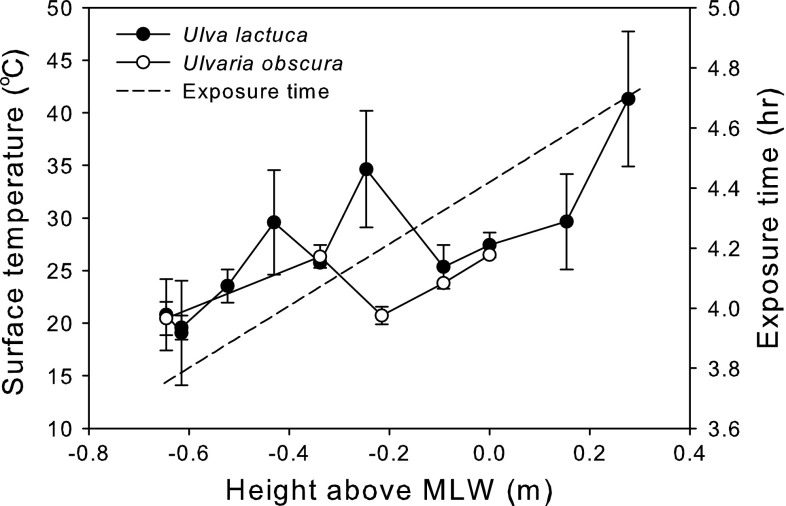
Mean surface temperatures (±1 SD, *N* = 1–9) of *U. lactuca* (*black circles*) and *U. obscura* (*open circles*) at different tidal heights immediately before they were immersed by an incoming tide. The *dashed* *line* indicates the amount of time that an alga would be exposed to the air during the low tide that occurred on the day that the measurements were taken based on estimates that were determined with Nobeltec Tides and Currents software

Surface temperatures of *U. lactuca* but not *U. obscura* differed among tidal heights at Ship Harbor (Fig. 5). Sixty-six of the 85 algal blades collected from Ship Harbor were determined to be *U. lactuca* by microscopic examination; the remaining 19 were *U. obscura*. *U. lactuca* was found at tidal heights ranging from 0.65 below MLW to 0.28 m above MLW, whereas *U. obscura*’s range was more limited, spanning 0.65–0.00 m below MLW. The surface temperatures of *U. lactuca* at Ship Harbor that were measured immediately prior to inundation by the incoming tide ranged from 18 to 47 °C and tended to increase with tidal height (Fig. 4; Spearman’s ρ = 0.735, *P* < 0.001, *N* = 66). *U. obscura*’s surface temperatures ranged from 19.5 to 27.8 °C, but there was no significant correlation between temperature and tidal height (Spearman’s ρ = 0.330, *P* = 0.168, *N* = 19).

**Fig. 6:**
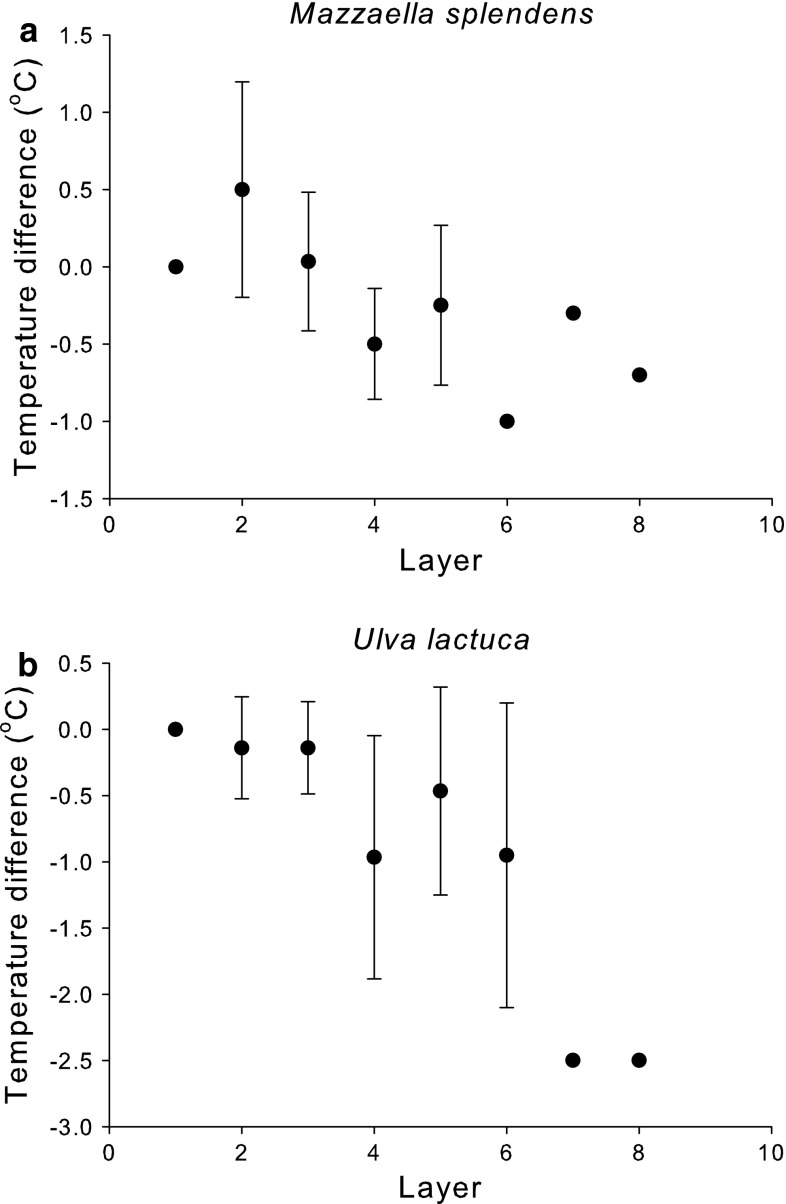
Average temperature difference (±1 SE; *N* = 1–10) between the measured layer and the top layer of **a** *M. splendens* and **b** *U. lactuca*

**Fig. 7:**
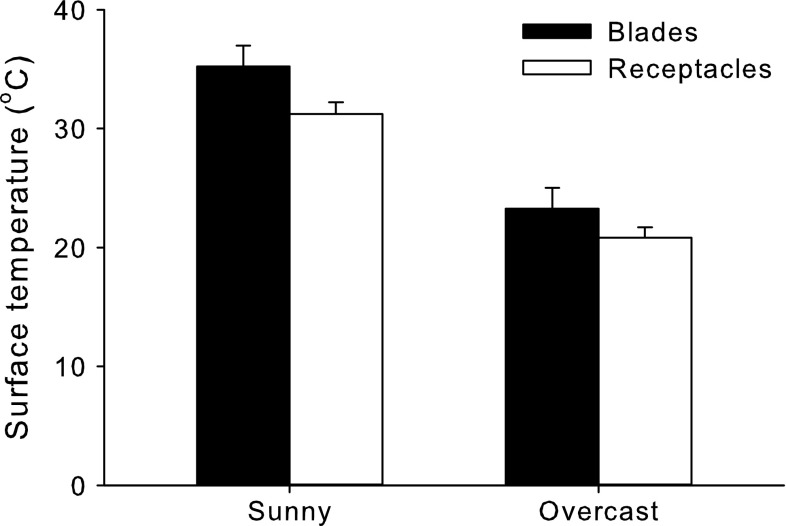
Average surface temperatures (± 1 SE; *N* = 5) of *F. distichus* blades (*black bars*) and receptacles (*white bars*) measured on a sunny and an overcast day

The surface temperatures of *M. splendens* and *U. lactuca* growing in stacks differed depending on where the algae were in the stack (Fig. 6). Algae in the lower layers were significantly cooler than algae in the top layers for both *M. splendens* (correlation: *R* ^2^ = −0.762, *P* = 0.028) and *U. lactuca* (correlation: *R* ^2^ = −0.916, *P* = 0.001). The surface temperatures of *F. distichus* receptacles were also about 3 °C cooler than the surfaces temperatures of blades (Fig. 7; two-way ANOVA: *df* = 1, *F* = 5.32, *P* = 0.035) and temperatures of both tissues were cooler on the overcast day than the sunny day (Fig. 7; two-way ANOVA: *df* = 1, *F* = 63.8, *P* < 0.001). There was no significant tissue × date interaction effect (Fig. 7; two-way ANOVA: *df* = 1, *F* = 0.32, *P* = 0.581). Surface temperatures of the gametophyte and crustose “*Petrocelis*” stages of *M. papillatus* were on average (±1 SE, *N* = 5) 26.4 ± 2.1 and 28.0 ± 1.1, respectively, and were not significantly different (*t* test: *df* = 8, *T* = 0.66, *P* = 0.53).

## Discussion

### Utility of the method and constraints

Infrared thermometers and cameras have been used in the past to measure surface temperatures of terrestrial plants (Fuchs and Tanner 1966; Idso et al. 1969; Berliner et al. 1984; Fuchs 1990; Hatfield 1990). They have occasionally been used to measure surface temperatures of intertidal invertebrates (Lee and Lim 2009; Lathlean et al. 2012; Chapperon et al. 2013), but only rarely have they been used to measure surface temperatures of intertidal seaweeds (Cox and Smith 2011). Previous measurements of emissivities of algal surfaces for tropical algae that were taken with an IR camera ranged from 0.98 to 0.99, slightly higher than the values that we obtained in this study, which were usually between 0.93 and 0.96 (Cox and Smith 2011). Our values were also similar to values obtained for leaves of terrestrial plants (Idso et al. 1969).

The intraspecific differences that we observed in emissivities were relatively small, with the exceptions of *D. viridis* and *C. exasperatus*. The lower and more variable emissivities that we observed in these species may be a result of their morphologies rather than their chemical compositions since other brown and red algal species, which would generally be similar in chemical composition within their respective groups, had higher and less variable emissivities. Most of the algal species that we examined were thin, flat blades that had relatively little surface texture. In contrast, *C. exasperatus* was covered with numerous papillae and *D. viridis* was composed of thin, cylindrical axes and branches. The papillae in *C. exasperatus* and the sides of the branches in *D. viridis* may have caused portions of the measurements to be taken from an angle, which is known to affect IR temperature readings (Fuchs et al. 1967). The differences in emissivities that we observed in *M. splendens* when the thermometer was angled at 90° versus 45° to the algal surfaces supports this hypothesis. These results contrast with the higher and more consistent estimates of emissivities of tropical algae obtained by Cox and Smith (2011). Some of the species that they examined, such as *Acanthophora spicifera* and *Laurencia mcdermidae*, were not flat blades, yet estimates of their emissivities made with an IR camera yielded values of about 0.99 with little variation. Whether the differences in our results and those of Cox and Smith (2011) are due to differences in the algal species used or differences in IR cameras versus IR thermometers is not known and merits further investigation.

The sensitivity analysis (Fig. 3) demonstrated that entering the wrong emissivity did not produce large errors in temperature readings for many macroalgal species, as long as the differences between the set and actual emissivities were relatively small. We also found that erring on the side of an emissivity setting that was too high had a smaller effect than operating the thermometer with an emissivity setting that was too low at all the temperatures tested. This result is in agreement with a similar analysis for IR cameras conducted by Cox and Smith (2011), which determined that setting the emissivity slightly above the actual emissivity resulted in errors that were generally <1 °C. However, moderate to large deviations from the correct emissivity can result in very erroneous readings at some temperatures including those within the range of temperatures that commonly occur at our study sites. Based on the results of this study, the use of IR thermometers to measure surface temperatures of many intertidal algae is warranted, with some caveats. For algae with thin flat blades that have relatively little surface texture, an emissivity setting of 0.94–0.97 provides results that are accurate to within about 1 °C. Actual emissivities and their variation should be determined empirically for algae that have a morphology that is not a flat blade or that has significant surface texture, such as papillae, in order to determine whether an IR thermometer can provide readings that are within the degree of accuracy required for the study. Our results also indicated that the degree of hydration and the presence of underlying layers of algae do not affect emissivities, although layering can affect the surface temperatures of algae in the underlying layers.

### Algal surfaces temperatures

High temperatures experienced by intertidal organisms at low tides in the summer tend to be more affected by the absorption of direct thermal radiation, heat conducted from the substratum to the organism, heat lost as a result of evaporation, and conductive losses of heat to the air than from the convection of heat from the air to the organism (Correa et al. 1986; Vadas et al. 1992; Denny et al. 2011). Consequently, intertidal organisms can attain temperatures at low tide that exceed air temperatures. Our measurements of maximum surface temperatures of intertidal algae were consistent with this. We found that surface temperatures of algae on the Shannon Point Beach (Fig. 4) generally exceeded air temperatures, especially in the mid intertidal zone and on the sunnier day.

The differences in surface temperatures of *U. lactuca* and *U. obscura* at Ship Harbor may be a result of the differences in the distributions of the two species and their abilities to tolerate desiccation. At nearby sites, *U. lactuca* (reported as *U. fenestrata*) has been documented to be more common in the intertidal zone, whereas *U. obscura* is more prevalent subtidally (Nelson et al. 2003). Thus, our measurements only included algae from the upper part of *U. obscura*’s distribution. *U. lactuca* also desiccates more slowly than *U. obscura* (Nelson et al. 2010), which may explain why we found it higher in the intertidal zone than *U. obscura.* Where the distributions of *U. obscura* and *U. lactuca* overlapped, their surface temperatures tended to be similar. The higher temperatures of *U. lactuca* in the high intertidal zone are likely due to the fact that it and the sand that it was on spent more time emersed and thus absorbed more solar radiation. It is unlikely that the differences in temperature were due to small-scale differences in surface heterogeneity as the site was a relatively homogeneous, shallow-sloped mud flat that had few topographic features.

Algae experience different temperatures at different stages of their life histories and younger stages are typically more vulnerable than older stages. In the central Salish Sea area, *F. distichus* produces fertile receptacles throughout the year, but reproduction peaks during the cooler fall and winter months (Ang 1991), which could be a mechanism to protect gametes from heat stress. Likewise, producing gametes in receptacles, which we showed were several degrees cooler than vegetative blades, may be another mechanism by which gametes are protected from thermal stresses.

Many algal species have heteromorphic biphasic or triphasic life histories that involve the alternation of a smaller crustose or microscopic stage and a larger upright stage (Lubchenco and Cubit 1980; Littler and Littler 1983; Fierst et al. 2010). The different phases are likely to have different physiological and ecological properties (Bell 1995). However, our surface temperature measurements demonstrated that the maximum temperatures experienced at low tide by the crustose and upright stages of *M. papillatus* were not significantly different on the Shannon Point Beach. Upright stages of *M. papillatus* may still be more prone to desiccation stress as desiccation rates are also affected by the degree to which the thallus is hydrated (Correa et al. 1986) and upright stages have more surface area exposed to air from which to lose water do than crusts. Further work is needed to determine whether the lack of differences in surface temperatures that we observed for the two life history phases of *M. papillatus* on the Shannon Point Beach also occurs at other locations and in other algal species with biphasic or triphasic life histories.

The distributions of individuals relative to one another can also affect the amounts of thermal stress that they experience. We found that the layering of *U. lactuca* and *M. splendens* affected algal surface temperatures, with thalli lower in the stack being cooler than algae higher up in the stack. We also found that the temperature differences between algae in the middle layers tended to be quite variable, which might be a result of differences in the amount of water retained between the layers in different stacks of algae. These interindividual differences are not surprising; interindividual differences in the maximum temperatures experienced by marine intertidal invertebrates have previously been documented (Harley 2008; Denny et al. 2011). Algal embryos, which tend to be more susceptible to the effects of environmental extremes than adults (Vadas et al. 1992), have also been shown to have higher survival rates under algal canopies than in exposed areas and within turfs, where temperatures were lower (Brawley and Johnson 1991).

In conclusion, an IR thermometer with the emissivity set to 0.95 or higher is an effective and rapid way to measure surface temperatures of intertidal algae that have relatively flat surfaces at fine scales (a few cm). However, before using an IR thermometer to measure surface temperatures of algae with papillate surfaces or species composed of thin axes and branches, it would be prudent to empirically determine the emissivity to ensure that the correct emissivity is being used. Both IR thermometers and cameras provide useful data about the temperatures attained by intertidal organisms. These measurements can be helpful in determining how environmental stresses impact these organisms, especially when the measurements are coupled with other measures of physiological stress (e.g., Smith and Berry 1986; Davison 1991; Bell 1993; Dethier et al. 2005; Williams and Dethier 2005).
